# Genomic profiling of DVL-1 and its nuclear role as a transcriptional regulator in triple negative breast cancer

**DOI:** 10.18632/genesandcancer.217

**Published:** 2021-10-13

**Authors:** Monica Sharma, Isabel Castro-Piedras, Austin Dwight Rodgers, Kevin Pruitt

**Affiliations:** ^1^Immunology and Molecular Microbiology, Texas Tech University Health Sciences Center, Lubbock, TX, USA

**Keywords:** Dishevelled (DVL), ChIP-Seq, transcription, histone modifications, breast cancer

## Abstract

Dishevelled-1 (DVL-1) mediates Wnt signals critical for development and cellular homeostasis. DVL-1 is also linked with tumorigenesis, however its association with specific breast cancer (BC) subtypes and how it contributes to tumorigenicity remains poorly studied. Herein, using bioinformatics and genomics analyses, we investigate the role of DVL-1 in different molecular subtypes of BC. Our results demonstrate that DVL-1 is highly expressed in triple-negative BC compared to non-cancer tissues and associated with various clinical factors that may contribute to poor prognosis and survival rate.

Another critical knowledge gap which remains poorly investigated involves the role of DVL-1 in the nucleus. While the cytoplasmic role of DVL-1 as a signaling hub has been extensively studied, the nuclear role of DVL-1 remains virtually unexplored. Herein for the first time, we have performed ChIP-Seq analyses to identify genomic regions targeted and regulated by DVL-1, thus highlighting its potential role as a regulator of transcription. Furthermore, we observed that DVL-1 peaks co-localize with H3K27me3 and EZH2, a repressive epigenetic mark and a histone methyltransferase respectively. Overall, our findings emphasize the importance of DVL-1 with TNBC-related pathology and identified unexpected gene targets of DVL-1, that may help explain the complexity of aberrant Wnt signaling in cancer.

## INTRODUCTION

Triple-negative breast cancer (TNBC) constitutes approximately 15–20% of breast cancers and is known for its aggressiveness and disproportionately higher rates of metastasis relative to other BC subtypes. It is also characterized by lack of estrogen receptors (ER), progesterone receptors (PR) and the absence of HER2 receptor overexpression on tumor cells, thereby limiting the use of available targeted therapies as a treatment option. Several reports suggest that oncogenic signaling mediated by the Wnt pathway is prominently associated with the TNBC-subtype [1]. Constitutive Wnt signaling is known to modulate the tumor microenvironment via fine tuning the crosstalk between malignant cells and infiltrating immune cells, making it an attractive therapeutic target in the treatment of TNBC [2]. DVL proteins provide a molecular hub through which Wnt signaling is relayed from upstream ligands and receptors to downstream effectors in order to control various cellular functions such as proliferation, stem cell renewal and migration [3]. DVL proteins have been classically associated with developmental disorders in a range of model organisms and with Robinow Syndrome in humans [3–5]. Emerging evidence suggests that DVL is also critical for breast tumorigenesis. Recently, our group showed that DVL-1 proteins are highly expressed in TNBC compared to non-cancer samples [6]. To further investigate its role, herein, we perform a comprehensive gene expression analysis of DVL-1 in different subtypes of breast cancer using the TCGA breast cancer datasets available in Tumor Immune Estimation Resource (TIMER), Human Protein Atlas (HPA), Breast Cancer Gen-ExMiner, UALCAN, and Kaplan Meier Plotter databases. Our results indicate that breast cancer tissues exhibit high levels of DVL-1 compared to normal counterparts. Moreover, we found DVL-1 to be significantly associated with various clinical risk factors for breast cancer patients such as cancer stage, menopausal status, ER, PR, HER2 receptor and triple-negative status. Interestingly, we also observed that high expression of DVL-1 is associated with poor overall survival rate in TNBC patients.

Another mystery surrounding DVL is its function in different subcellular compartments. DVL has been studied primarily as a cytoplasmic scaffold which promotes either β-catenin stabilization or cell migration [7, 8]. However, seminal studies show that DVL proteins shuttle between the cytoplasm and nucleus, which is essential for Wnt/β-catenin signaling [9–11]. Moreover, our group was the first to report novel post-translational lysine acetylation of DVL proteins which acts as a regulatory switch to promote its nuclear localization [6]. Limited reports demonstrated that DVL binds to the promoters of Wnt target genes such as cMyc, BMP4, and cyclinD1 [11, 12]. Moreover, our previous study extended this binding repertoire and demonstrated that DVL proteins bind and regulates genes such as *CYP19A1*, a target not previously recognized as a Wnt target gene [13]. For a more comprehensive analysis of DVL nuclear function, we screened for novel DVL-1 target genes and discovered that DVL-1 binds to various genes involved in cancer biology and T-cell mediated immunity in TNBC models. Furthermore, analysis using TIMER database and gene expression studies indicates that high levels of DVL-1 are inversely correlated with target genes including infiltration of CD8+ and CD4+ T cells, highlighting a potential connection of DVL-1 in cancer immunomodulation.

Collectively, our findings provide additional insights into the contribution of DVL-1 in triple-negative breast tumorigenesis as well as suggests that nuclear DVL-1 could modulate tumor microenvironment by transcriptionally regulating genes involved in various cellular functions. Therefore, these findings propose DVL-1 as a promising prognostic biomarker for triple-negative tumors, a breast cancer subtype which urgently needs effective treatment options.

## RESULTS

### DVL-1 is highly expressed in various tumor types including breast cancer

**Figure 1:**
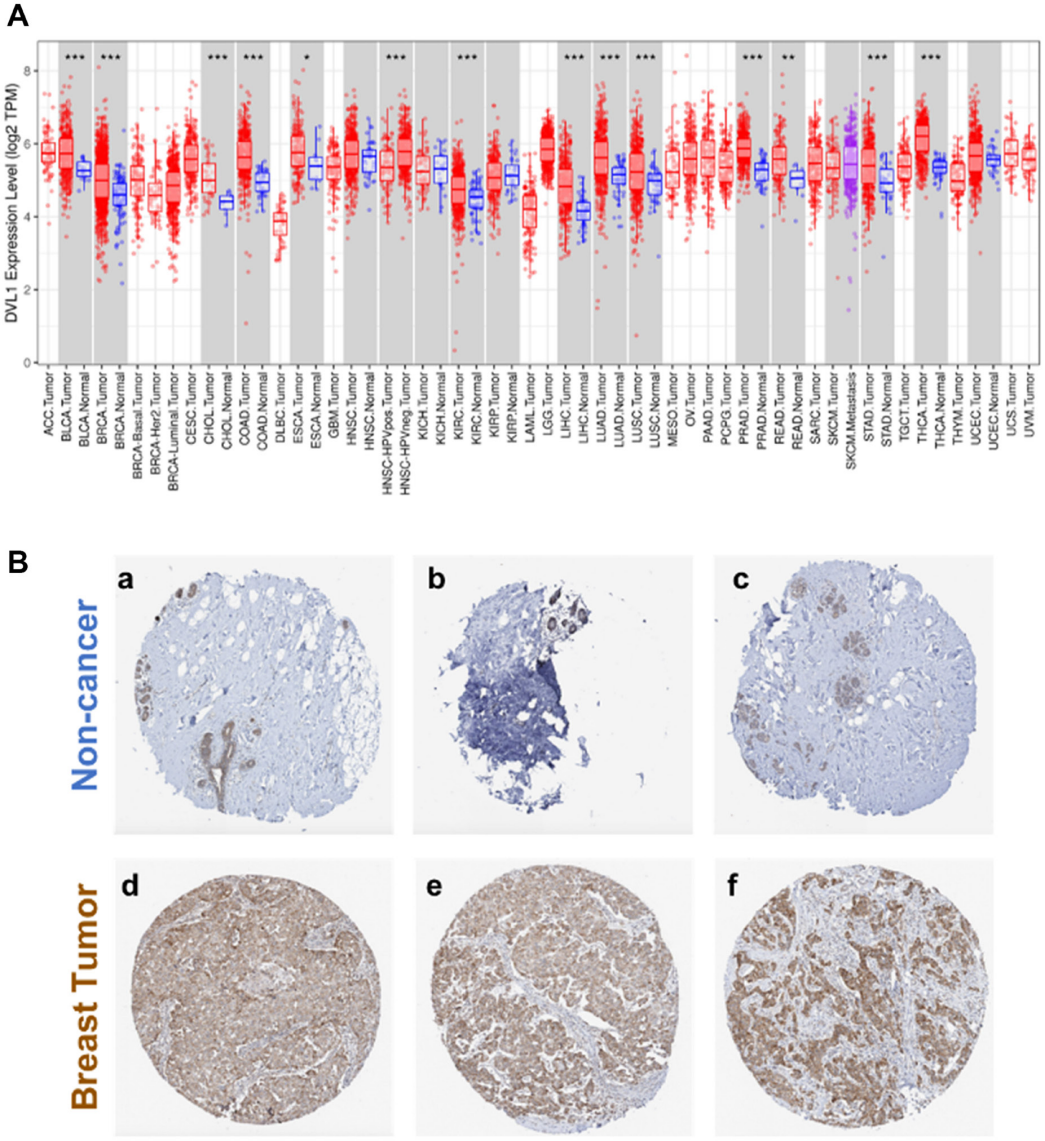
Expression of human Dishevelled-1 (DVL1) in different tumor types. (**A**) DVL-1 expression levels in different tumor types from The Cancer Genome Atlas (TCGA) database were determined using Tumor Immune Estimation Resource (TIMER). Compared with normal tissues (in blue color), DVL-1 was upregulated in various cancer types (in red color) such as bladder urothelial carcinoma (BLCA), breast cancer (BRCA), cholangiocarcinoma (CHOL), colon adenocarcinoma (COAD), esophageal carcinoma (ESCA), kidney renal cell carcinoma (KIRC), liver hepatocellular carcinoma (LIHC), lung adenocarcinoma (LUAD), lung squamous cell carcinoma (LUSC), prostate adenocarcinoma (PRAD), rectum adenocarcinoma (READ), stomach adenocarcinoma (STAD), and thyroid carcinoma (THCA). **(B)** DVL-1 protein expression in breast cancer tissues and normal tissues from the Human Protein Atlas (HPA) database. Immunohistochemical staining revealed that DVL-1 exhibited low expression in normal (adjacent) tissues (a–c) versus high expression in breast tumor tissues (d–f). *P*-value Significant Codes: 0 ≤ ^***^ < 0.001 ≤ ^**^ < 0.01 ≤ ^*^ < 0.05 ≤ . < 0.1.

Dishevelled (DVL) proteins regulate oncogenic Wnt signaling which is a major driver of tumor progression [3]. Since DVL relays oncogenic input signals, we hypothesized that the expression levels of DVL-1 would be high in various cancer types. In order to understand the differential expression of DVL-1 between various tumor types (in red color) and its adjacent normal tissues (in blue color), we analyzed RNA-Seq data from TCGA. As shown in Figure 1A, using the TIMER database we found that DVL-1 was significantly upregulated in various cancer types which have hyper-activated Wnt signaling including breast cancer, colon adenocarcinoma, liver hepatocellular carcinoma, lung adenocarcinoma, prostate adenocarcinoma compared to adjacent normal (healthy) tissues [14, 15]. In addition, to further validate the expression of DVL-1 protein in patient tissues, we used the pathology images from the HPA database, where DVL-1 is stained in normal and cancer tissues using antibody CAB011538. As presented in Figure 1B, the levels of DVL-1 antibody staining in three cases of breast cancer were high compared to the non-cancer adjacent tissues. Since the expression of both DVL-1 transcript and protein was significantly higher in breast tumor tissues, we next evaluated its association with different clinical parameters in breast cancer patients.

### Association of DVL-1 expression with various clinical parameters and overall survival in breast cancer

**Figure 2:**
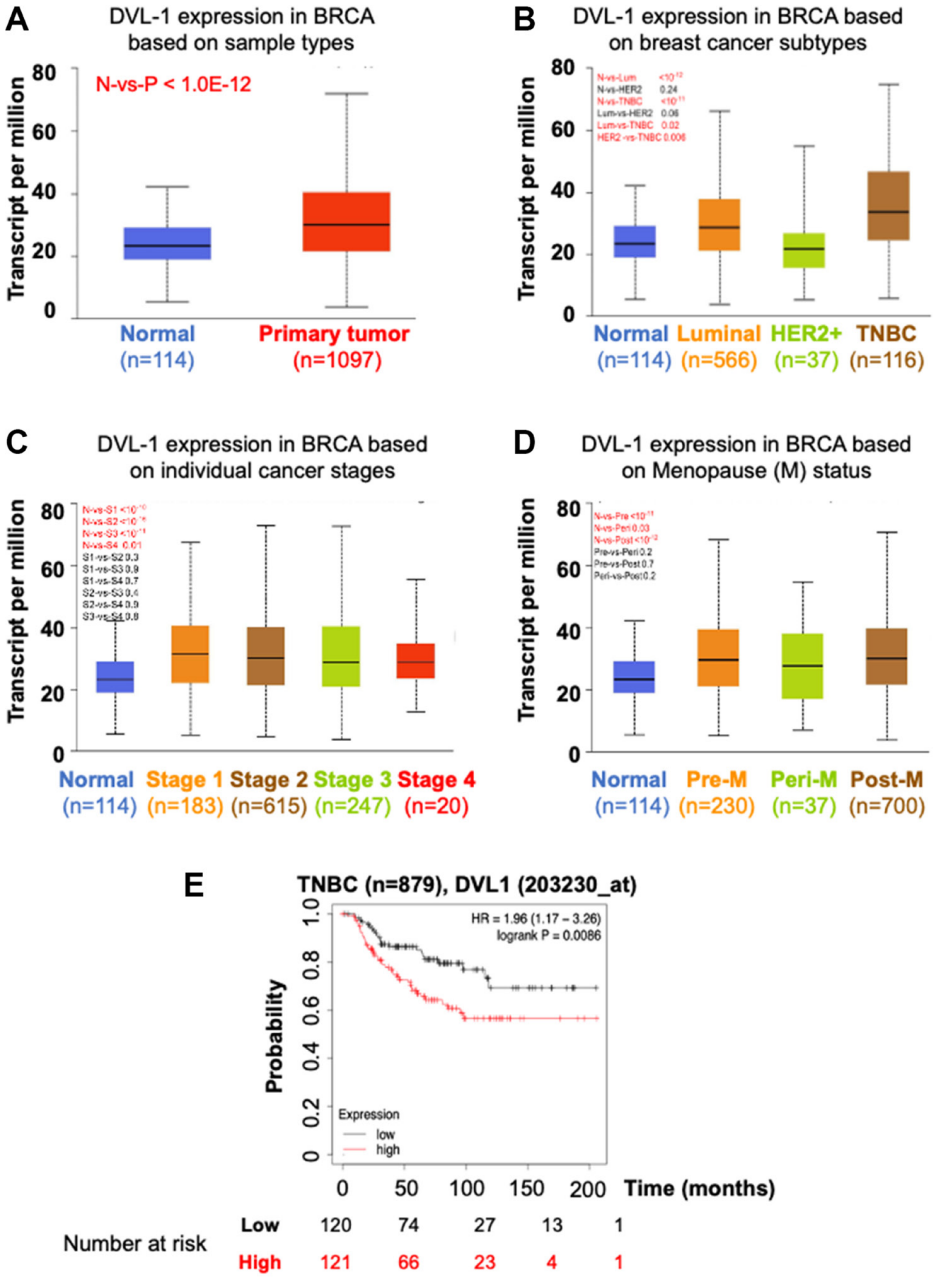
Association between DVL-1 expression, clinicopathological parameters and overall survival (OS) in different breast cancer subtypes. UACLAN analysis is performed using TCGA data to evaluate the correlation between DVL-1 expression based on (**A**) Sample type – normal (N, *n* = 114) and primary tumor (P, *n* = 1097). (**B**) Breast cancer subclasses – normal (N, *n* = 114), luminal (Lum, *n* = 566), HER2 positive (HER2, *n* = 37), and triple-negative (TNBC, *n* = 116). (**C**) Individual cancer stages – stage 1 (S1, *n* = 183), stage 2 (S2, *n* = 615), stage 3 (S3, *n* = 247), and stage 4 (S4, *n* = 20). (**D**) Menopause status – normal (N, *n* = 114), pre-menopause (Pre, *n* = 230), peri-menopause (Peri, *n* = 37), and post-menopause (Post, *n* = 700). (**E**) Kaplan Meier database was used to compare survival rates between high and low expression of DVL1 (203230_at) in triple-negative breast cancer.

We assessed DVL-1 patterns in different molecular subtypes along with various clinical pathological parameters of breast cancer patients such as sample types, tumor stages, and menopausal status. Using TCGA breast cancer data cohort from UALCAN analysis platform, we confirmed that the expression of DVL-1 in breast cancer samples (*n* = 1097) was significantly higher than that in non-cancer/normal cancer tissues (*n* = 114) with a *p*-value <10^−12^ (Figure 2A). Next, the analysis based on different subtypes of breast cancer showed that expression of DVL-1 was significantly higher in luminal group (*n* = 566, *p* < 10^−12^) and triple-negative group (*n* = 116, *p* < 10^−11^) compared to normal control group. We observed that there was no statistical difference in the HER2+ subtype (*n* = 37, *p* < 0.24) of breast cancer compared to normal group. Interestingly, DVL-1 expression was significantly higher in triple negative subtype compared to other molecular subtypes including luminal (*p* < 0.02) and HER2+ (*p* < 0.006) groups (Figure 2B). Moreover, all stages of breast cancer had a significantly higher DVL-1 expression relative to the normal group. However, there was no significant difference in DVL-1 expression between the stages (Figure 2C). Expression of DVL-1 was also significantly higher in different classification of menopausal status: pre-menopausal (*p* < 10^−11^), peri-menopausal (*p* < 0.03) and post-menopausal status (*p* < 10^−12^) compared to normal (Figure 2D). Furthermore, we explored the correlation between DVL-1 mRNA levels and different risk factors such as lymph nodal status, ER status, PR status, HER2 status, and triple-negative status using a different database system called BC-GenExMiner 4.4 (Table 1). We observed that there was no difference in DVL-1 mRNA expression between nodal positive versus nodal negative status. However, based on the hormone receptor status, there was no significant different between ER-positive and ER-negative samples. However, DVL-1 mRNA levels were significantly highly expressed in PR-negative (*p* = 0.01), HER2-negative (*p* = 0.01) and triple-negative status (*p* = 0.004), further substantiating the idea that DVL-1 expression may serve as a potential diagnostic biomarker for specific subtypes of breast cancer such as triple-negative breast cancer.

**Table 1:** Relationship between mRNA expression of DVL-1 and clinicopathological parameters of breast cancer

Variables	DVL-1	
	*N*	*P*-value
**Nodal Status**		
−	402	0.06
+	479	
**Estrogen receptor status (ER)(IHC)**		
−	228	0.05
+	756	
**Progesterone receptor status (PR)(IHC)**		
−	325	0.01
+	656	
**HER2 receptor status (HER2)(IHC)**		
−	532	0.01
+	155	
**Triple-negative status (TNBC)(IHC)**		
TNBC	111	0.004
Not TNBC	810	

BC-GenExMiner 4.4 was used to explore the correlation between DVL-1 mRNA expression and common factors such nodal status (negative and positive), estrogen receptor (ER) status (negative and positive), progesterone receptor (PR) status (negative and positive), HER2 receptor status (negative and positive), and triple-negative status (TNBC vs. not-TNBC).

Since the bioinformatics analyses showed promising results suggesting DVL’s association with various clinical risk factors for breast cancer, we wanted to explore the correlation between gene amplification of DVL-1 and patient survival. Using Kaplan Meier survival curves, we compared whether different expression levels of DVL-1 has any association with overall survival (OS) in triple-negative breast cancer patients. Notably, we observed that high DVL-1 was significantly associated with lower overall survival, hence poor prognosis in TNBC patients. The hazard ratio was significantly higher in TNBC group with a *p*-value equivalent to 0.009 (Figure 2E). Moreover, the results indicate that there was no significant association between DVL-1 expression and survival rates in luminal A/B, and HER2+ breast cancer subtypes (Supplementary Figure 1). These findings show that DVL-1 is significantly associated with mortality risk, suggesting that DVL-1 could serve as a prognostic marker for TNBC patients.

### DVL-1 binds to different genomic locations in breast cancer models

**Figure 3:**
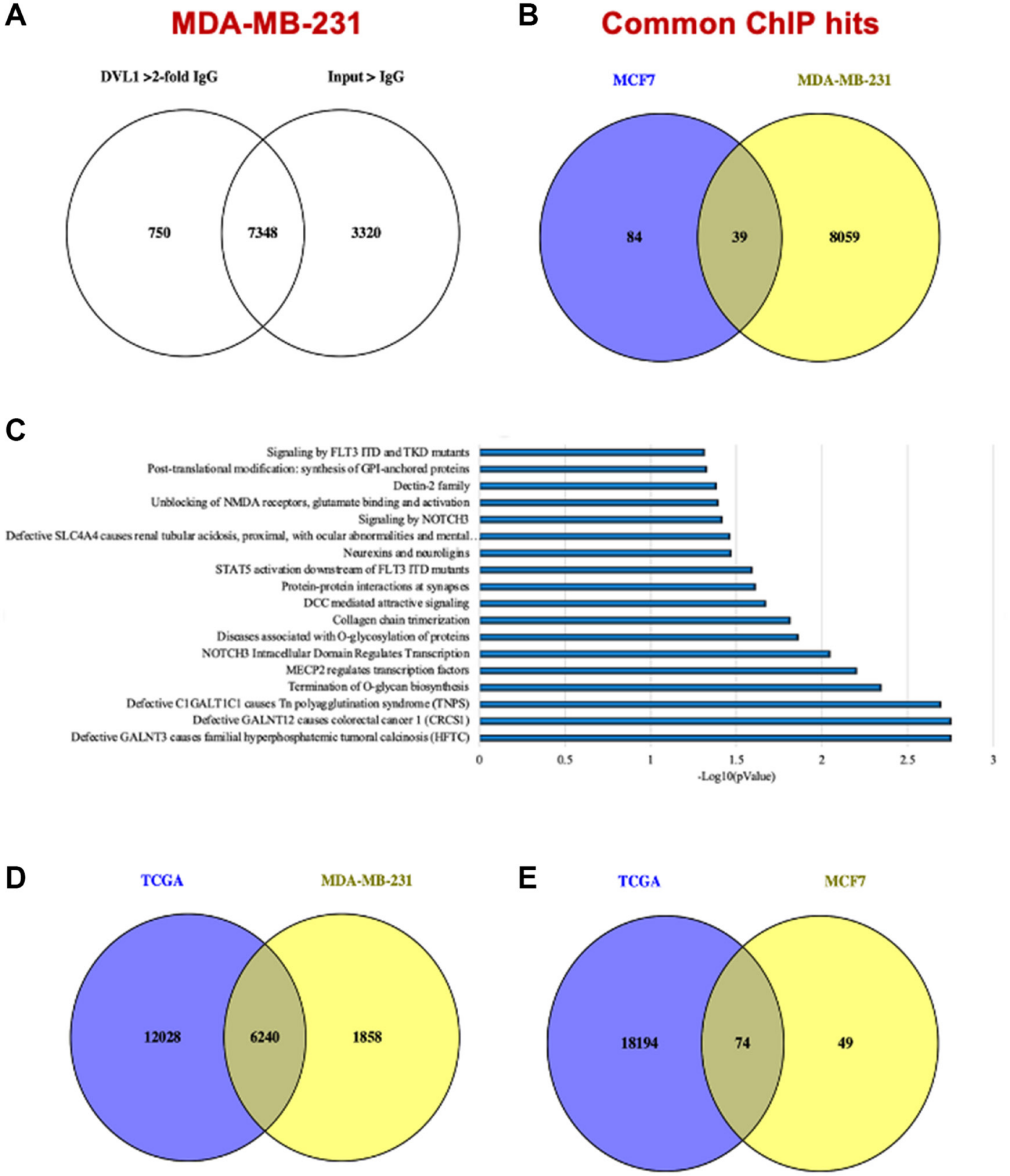
DVL-1 localizes at 8000 different genomic locations in breast cancer models. (**A**) Venn diagram representing DVL-1 ChIP-Seq peaks over IgG (≥2-fold ) and Input control. (**B**) Venn diagram representing DVL-1 ChIP-Seq peaks overlapping between MDA-MB-231 and MCF7 cells. (**C**) Table showing enriched pathways of ChIP-hits for MDA-MB-231 and MCF7 cell lines generated by Reactome pathway analysis. (**D**) Venn diagram representing overlap between MDA-MB-231 DVL-1 ChIP-Seq peaks and genes altered in TCGA breast cancer patient data. (**E**) Venn diagram representing overlap between MCF7 DVL-1 ChIP-Seq peaks and genes altered in TCGA breast cancer patient data.

DVL plays a critical role in relaying Wnt signals within the cytoplasm for cellular growth, however its role in the nucleus remains virtually unexplored. Intrigued by the recent discoveries made by our group regarding the identification of a “regulatory switch” that controls nuclear translocation [6], we performed three independent DVL-1 ChIP-seq analyses in MDA-MB-231 and MCF7 cells to evaluate the role of nuclear DVL-1. For the ChIP-Seq experiments we used an IgG-control ChIP-Seq dataset as a reference for peak selection which showed a robust reproducibility between experiments in both cell lines. We obtained a total of 8098 peaks for DVL-1 in MDA-MB-231 cells (≥2-fold enrichment over IgG, intersecting 7348 Input genes), and a total of 123 peaks for DVL-1 in MCF7 (≥2-fold enrichment over IgG). Interestingly, 39 of the filtered MCF7 peaks overlapped with MDA-MB-231 peaks (Figure 3A–3B). Furthermore, we performed pathway analysis using the Reactome database which indicated that DVL-1 gene hits were involved in various biological processes including signaling transduction, post-translational modifications, protein-protein interactions, and human-associated diseases (Figure 3C). To investigate whether these findings could also be linked with breast cancer patients, we analyzed RNA-Seq data from 1904 breast cancer patients using cBioportal database (TCGA analysis). The RNA-Seq data was downloaded from Breast cancer study (Metabric, Nature 2012 & Nat Comm 2016), where about 18,268 genes were altered in primary breast tumors [16]. We found that there was an overlap of 6,240 genes and 74 genes altered in breast cancer patients and DVL-1 ChIP Seq hits in MDA-MB-231 and MCF7 cells respectively, which corresponds to 31% of DVL-1 hits in MDA-MB-231 and 0.4% of DVL-1 hits in MCF7 (Figure 3D–3E), further suggesting the importance of subtype-specific DVL-1 associations in patient tumors.

### DVL-1 localizes at cancer-associated genes and regulates their expression

**Figure 4:**
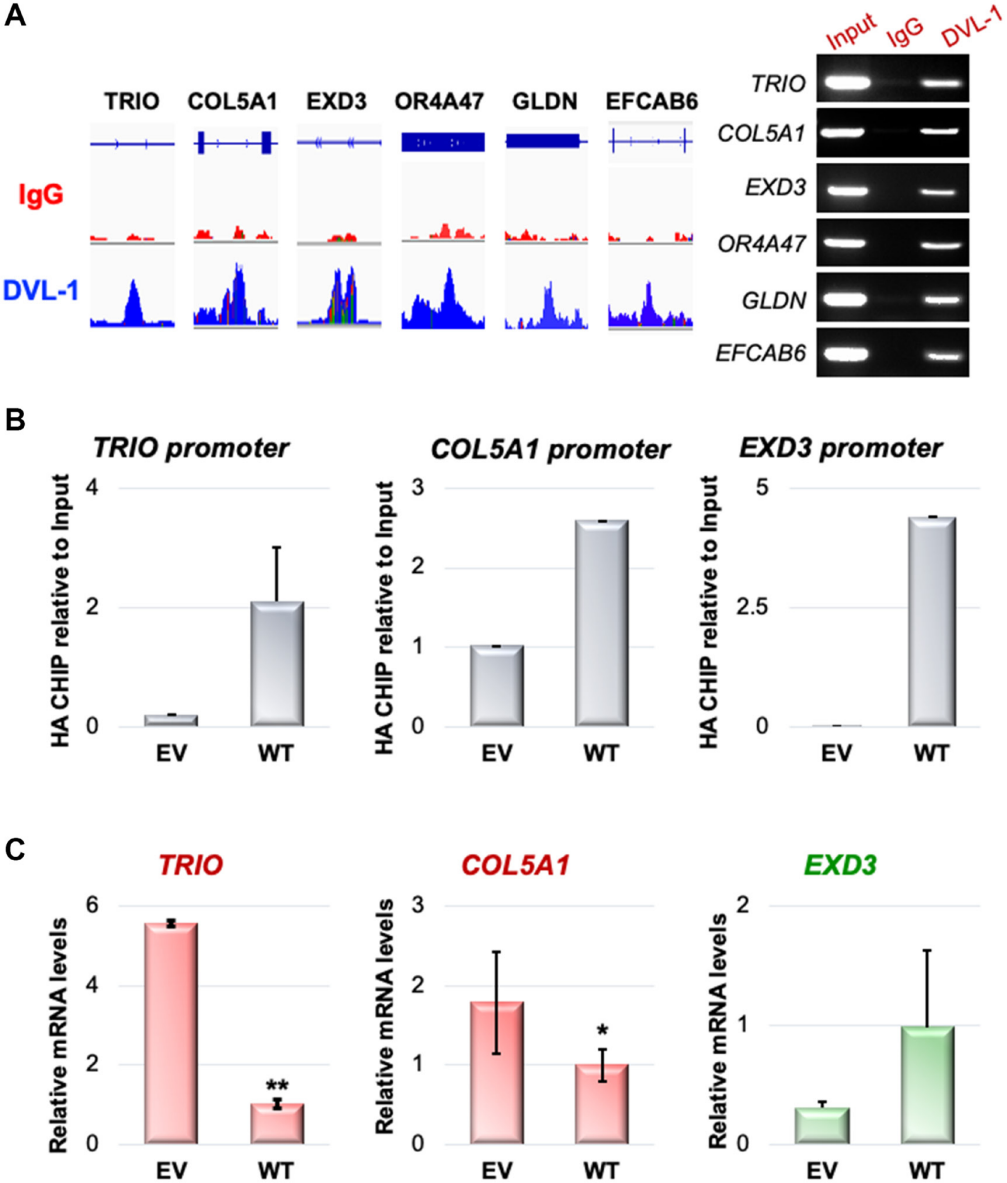
DVL-1 localizes at cancer-associated genes and regulates their expression in MDA-MB-231 cells. (**A**) An assembly of IgG (first row) and DVL-1 (second row) ChIP-Seq data in MDA-MB-231 for the *TRIO, COL5A1, EXD3, OR4A47, GLDN*, and *EFCAB6* genes, visualized by IGV. On the right, ChIP for species-matched IgG and DVL-1 followed by PCR analysis for Input and ChIP samples at the indicated genes in MDA-MB-231 cells. (**B**) ChIP-qPCRs at *TRIO, COL5A1, EXD3* genes for HA-tag in MDA-MB-231 (EV and WT-DVL1) (**C**) RT-qPCR-based analysis of expression changes of *TRIO, COL5A1, EXD3* genes in MDA-MB-231 (EV and WT-DVL1) cells. Transcript levels were normalized to actin transcript levels.

We previously reported that DVL proteins not only localize to the nucleus of breast cancer cells, but it also binds to *CYP19A1* promoters and regulates its transcription [13]. Using the ChIP Seq bam files, we visualized the exact genomic location of DVL-1 binding (blue peak) at various cancer-associated genes not previously designated as Wnt target genes such as *TRIO, COL5A1, EXD3, OR4A47, GLDN*, and *EFCAB6* compared to IgG control (red peak) using IGV (Figure 4A, Supplementary Figure 2). Next, we designed ChIP primers that spanned DVL-1 binding location on multiple genes and validated DVL occupancy by ChIP-PCR in MDA-MB-231 cells (Figure 4B). Additionally, we performed mRNA expression analysis to determine DVL-1 mediated regulation of target genes in MDA-MB-231 cells. Using RT-qPCR, we observed a reduction in the mRNA expression of *TRIO* and *COL5A1* genes in WT (wild-type) MDA-MB-231 cells overexpressing DVL-1 compared to EV (empty vector) control cells. We tested the over-expression of DVL-1 on another gene, *EXD3*, however there does not appear to be a significant change in *EXD3* expression with DVL-1 over-expression, suggesting a multidimensional role of DVL-1 that may depend on the genomic context (Figure 4C). These results clearly demonstrate that DVL-1 binds to multiple novel target genes and regulates the mRNA expression of some of its target genes we tested here.

### DVL-1 localizes to genes crucial for immune regulation and inversely regulates infiltrating T cell genes

**Figure 5:**
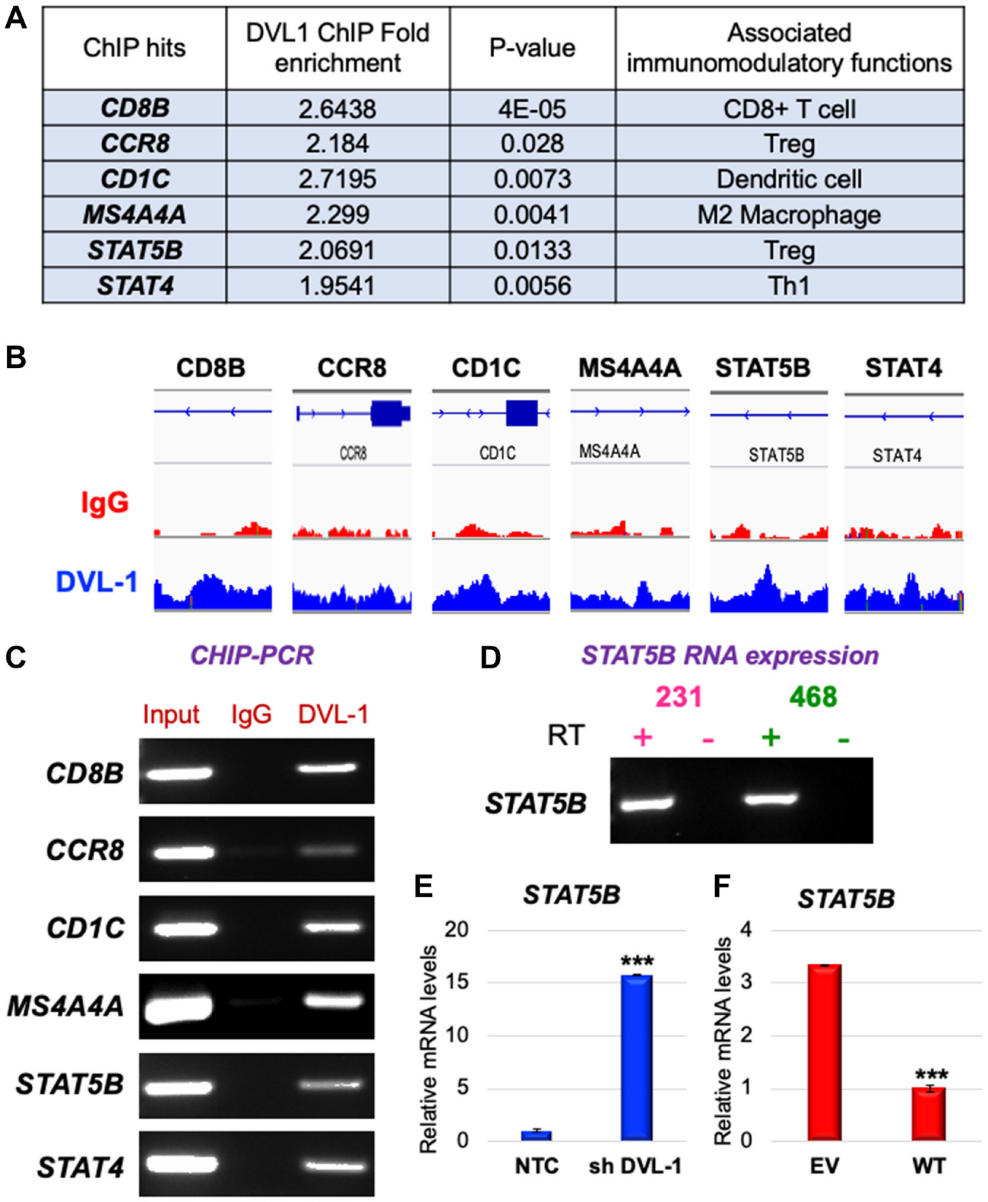
DVL-1 localizes to genes of immune cell genes and regulates its expression. (**A**) List of genes bound by DVL-1 at genomic regions identified using ChIP sequencing analyses in MDA-MB-231 cells. (**B**) Assembly of IgG (first row, red peak) and DVL-1 (second row, blue peak) ChIP-Seq data in MDA-MB-231 for CD8B, CCR8, CD1C, MS4A4A, STAT5B, and STAT4 genes, visualized by IGV. (**C**) ChIP RT-PCR at different genes for Input, IgG and DVL-1 in MDA-MB-231 cells. (**D**) mRNA expression analysis of the *STAT5B* gene to determine its expression patterns in MDA-MB-231 and MDA-MB-468 cells using end-point RT-PCR. RT-qPCR-based analysis of STAT5B gene expression changes in MDA-MB-231 cells stably expressing (**E**) NTC vs. shDVL-1 and (**F**) EV vs. WT-DVL1. Transcript levels were normalized to actin transcript levels. For statistical analysis, independent *t* test was performed where ^***^p < 0.001.

In our ChIP-Seq screen, we discovered that DVL-1 localizes at genomic regions critical for immune regulation as listed in Figure 5A. We used IGV to visualize the exact genomic location of DVL-1 binding (blue peak) at different genes compared to IgG control (red peak) as presented in Figure 5B. We designed ChIP primers that spanned DVL-1 binding location on multiple lymphocyte genes and then validated DVL occupancy at *CD8B, CCR8, CD1C, MS4A4A, STAT5B*, and *STAT4* genes by ChIP-PCR in MDA-MB-231 cells (Figure 5C, Supplementary Figure 3). Additionally, we performed mRNA expression analysis to determine the expression of DVL-1 targets in MDA-MB-231 cells. Interestingly, we observed that out of 6 genes only *STAT5B* mRNA was detected in TNBC cells, suggesting that DVL-1 may likely bind to transcriptionally repressed genes (Figure 5D). Furthermore, we wanted to evaluate whether DVL-1 regulates target genes such as *STAT5B*, which has been shown to play a critical role in cancer and anti-tumor immunity [17]. Using quantitative RT-PCR, we found that knockdown of DVL-1 resulted in upregulation of *STAT5B* expression, suggesting that DVL-1 acts as a repressor of *STAT5B* (Figure 5E). In contrast, over-expression of WT-DVL1 resulted in a significant reduction in *STAT5B* expression (Figure 5F), further validating that DVL-1 could potentially modulate anti-tumor immune responses by localizing to different genomic sites and inversely regulating expression of genes critical for anti-tumor immunity.

To further understand the relationship between DVL-1 expression and different immune cell populations, we used the TIMER database to examine the correlation between DVL-1 expression and genes expressed by different lymphocyte subsets such as CD4+ T helper cells (e.g. Th1, Th2 and Th17), CD4+ regulatory T cells (Treg), follicular helper T cells (Tfh), CD8+ T cell and B cells. In addition, we used the TIMER database to determine the correlation between DVL-1 expression and genes expressed by different innate immune cells including monocytes, tumor-associated macrophages (TAM), M1/M2 macrophages, neutrophils, NK cells and dendritic cells in luminal, HER2-positive, and TNBC subtypes of breast cancer as summarized in Table 2 and Supplementary Figure 4. In our screen, we analyzed the immune cell genes that significantly correlated with DVL-1 expression with a *p*-value less than 0.05 (as highlighted in Table 2). We found that expression of DVL-1 was significantly correlated with immune cell genes in different breast cancer subtypes (Luminal, *n* = 611; HER2-positive, *n* = 67; and TNBC, *n* = 139). Particularly, the expression levels of DVL-1 was negatively and significantly correlated with 16 immune cell gene markers in TNBC breast cancer, such as *CD2, CD86, CCL2, CD68, IL10, MS4A4A, KIR3DL2, HLA-DRA, HLA-DPA1, STAT4, STAT1, GATA3, IL21, CCR8, CTLA4, and HAVCR2*. Interestingly, expression of some immune cell genes and DVL-1 uniquely correlated with luminal breast cancer, such as *CD19, CD79A, CD8B, CCR7, CD1C,* and *STAT5B*.

**Table 2:** Correlation between DVL-1 expression and markers of immune cells in breast cancer

DESCRIPTION	GENE MARKERS	BRCA-LUMINAL (*N* = 611)		BRCA-HER2 (*N* = 67)		BRCA-TNBC (*N* = 139)	
		Cor	*p*-value	Cor	*p*-value	Cor	*p*-value
B cell	CD19	0.0046597	0.913494164	0.04140914	0.75549825	–0.2261982	**0.00994861**
	CD79A	–0.009124	0.831542125	–0.0369959	0.780375818	–0.228835	**0.00922312**
T cell	CD3D	–0.064502	0.132244282	0.00362361	0.978376301	–0.1580445	0.07368264
	CD3E	–0.080455	0.060284068	–0.02782	0.83398407	–0.1568426	0.075923183
	CD2	–0.113064	**0.008184703**	–0.0606078	0.647581519	–0.1818146	0.039320345
CD8+ T cell	CD8A	–0.03664	0.392840297	–0.0257744	0.84604819	–0.1445886	0.10204675
	CD8B	–0.034776	0.417378575	0.03395675	0.798031544	–0.1904908	**0.030732045**
Monocyte	CD86	–0.184081	**1.50E-05**	–0.1161309	0.380095014	–0.1938562	**0.027859995**
TAM	CCL2	–0.157796	**0.000213974**	–0.1717709	0.192803141	–0.1507156	0.088214723
	CD68	–0.128764	**0.002574535**	–0.1036821	0.433507679	–0.1358844	0.1245725
	IL10	–0.168813	**7.37E-05**	–0.1654004	0.210021842	–0.1524877	0.084506427
M1 Macrophage	NOS2	–0.042345	0.323336377	0.16306254	0.21660626	–0.0284018	0.749342348
	IRF5	–0.017485	0.683516701	–0.0199299	0.880703304	0.02967352	0.738194431
M2 Macrophage	CD163	–0.032746	0.445096892	–0.0987142	0.455954581	–0.1455557	0.099756256
	VSIG4	–0.046773	0.2752668	–0.1527177	0.247486008	–0.1207122	0.17277137
	MS4A4A	–0.17308	4**.79E-05**	–0.1997078	0.129210713	–0.1813953	**0.039782018**
Neutrophils	CEACAM8	–0.071667	0.094339597	–0.2079493	0.1140052	0.09022021	0.309245703
	ITGAM	–0.074594	0.081605213	–0.0712449	0.590899586	–0.0992621	0.262681878
	CCR7	–0.016194	0.705759969	0.12893045	0.329528208	–0.2000503	**0.023170596**
Natural killer cells	KIR2DL1	–0.060979	0.154757284	–0.018518	0.889285542	–0.0911521	0.30425437
	KIR2DL3	–0.056307	0.188932789	0.11967614	0.366615011	–0.0386566	0.663603305
	KIR3DL1	–0.039552	0.356299066	–0.0896774	0.499389981	–0.0798297	0.368491565
	KIR3DL2	–0.086955	**0.042250171**	–0.1985213	0.131736054	–0.0858284	0.333483855
	KIR3DL3	0.0081237	0.849785527	–0.1086901	0.412539191	–0.0930139	0.2944418
	KIR2DS4	0.0030185	0.943898316	–0.0161586	0.903319451	–0.0453363	0.609931269
Dendritic cells	HLA-DPB1	–0.009933	0.816870427	0.07083577	0.593037817	–0.1180344	0.182549654
	HLA-DQB1	–0.04385	0.30641251	–0.1990064	0.130582081	–0.131032	0.138700884
	HLA-DRA	–0.139387	**0.001092725**	–0.0933372	0.480955449	–0.2016156	**0.022099154**
	HLA-DPA1	–0.144852	**0.00068664**	–0.1326125	0.315812219	–0.2183699	**0.01305403**
	CD1C	–0.035466	0.408186814	–0.0167787	0.899628151	–0.1750478	**0.047238406**
	ITGAX	–0.061916	0.14850528	–0.0524839	0.692286752	–0.157178	0.075292444
Th1	TBX21	–0.012384	0.77279054	0.00233781	0.986110628	–0.1281138	0.147767936
	STAT4	–0.13986	**0.001050299**	–0.0388662	0.769565062	–0.2236583	**0.010972081**
	STAT1	–0.171199	**5.80E-05**	–0.1323203	0.316887168	–0.1865105	**0.034450435**
	IFNG	–0.08096	0.058685485	–0.0765676	0.564351067	–0.1353733	0.126108217
	TNF	–0.015774	0.713045283	–0.0979544	0.459443231	0.00626677	0.943753154
Th2	GATA3	0.1219274	**0.004328742**	0.31367621	0.015894689	0.01678779	0.850042493
	STAT6	0.0940068	**0.028059394**	–0.051315	0.698811092	–0.0821165	0.354419627
	STAT5A	0.0930258	**0.029747692**	–0.0558738	0.673493808	–0.0993683	0.262169585
	IL13	–0.046235	0.280832776	0.12899372	0.330207686	–0.0797132	0.369193244
Tfh	BCL6	0.0373671	0.383509691	–0.1253653	0.34316356	–0.0040977	0.963212664
	IL21	–0.104767	**0.014317684**	–0.1158771	0.382132421	–0.1251018	0.157770632
Th17	IL17A	–0.007988	0.852266864	–0.074007	0.577486223	–0.0743762	0.402205084
Treg	FOXP3	–0.059286	0.166554166	–0.018761	0.887663456	–0.1243907	0.15997923
	CCR8	–0.135538	**0.001501096**	–0.0609585	0.645678164	–0.1927225	**0.028658177**
	STAT5B	0.0513082	0.231329692	–0.0804793	0.54358993	–0.2325973	**0.008111482**
	TGFB1	0.045913	0.284197029	0.24003507	0.067250011	–0.0750671	0.397345376
Tcell exhaustion	PDCD1	0.0406328	0.343298604	0.10829924	0.413218902	–0.0703797	0.428032756
	CTLA4	–0.112693	**0.00839852**	–0.0345412	0.794628038	–0.1629025	0.065166522
	HAVCR2	–0.202982	**1.74E-06**	–0.1271186	0.336414085	–0.1977862	**0.024799287**
	GZMB	–0.0604	0.158714516	–0.0142022	0.914884465	–0.1138363	0.198957762

Investigating the correlation between expression of DVL-1 with immune infiltration levels in breast cancer using TIMER. Bold values indicate the *p*-value < 0.05 with moderate correlation. RNA-Seq data from TCGA database is used to detect and quantify the immune infiltration levels in breast tumor subtypes.

Interestingly, we found that DVL-1 mRNA expression is inversely correlated with B cell antigen (*CD19)*, T-cell co-receptor (*CD8A*, *CD8B)*, transcription factors that play a major role in innate and adaptive immune response (*STAT1, STAT4)*, human leucocyte antigens (HLA) genes that are critical for encoding the major histocompatibility complex (MHC) II genes (*HLA-DRA, HLA-DPA1)*, inflammatory cytokines (*IFNG*) and lymphocyte chemokine receptors (*CCR7)*. Importantly, we found that the immunosuppressive cytokine, IL-10 was positively associated with DVL-1 mRNA expression in breast cancer patients. Taken together, these results suggest that there may be an important link between DVL-1 expression in breast cancer cells and the suppression of T cell mediated anti-tumor responses, perhaps by transcriptionally repressing the expression of various immune cell-related genes.

### Nuclear DVL-1 binding is enriched on specific motif sequences and localizes with histone methyltransferase EZH2 and H3K27me3 epigenetic mark

**Figure 6:**
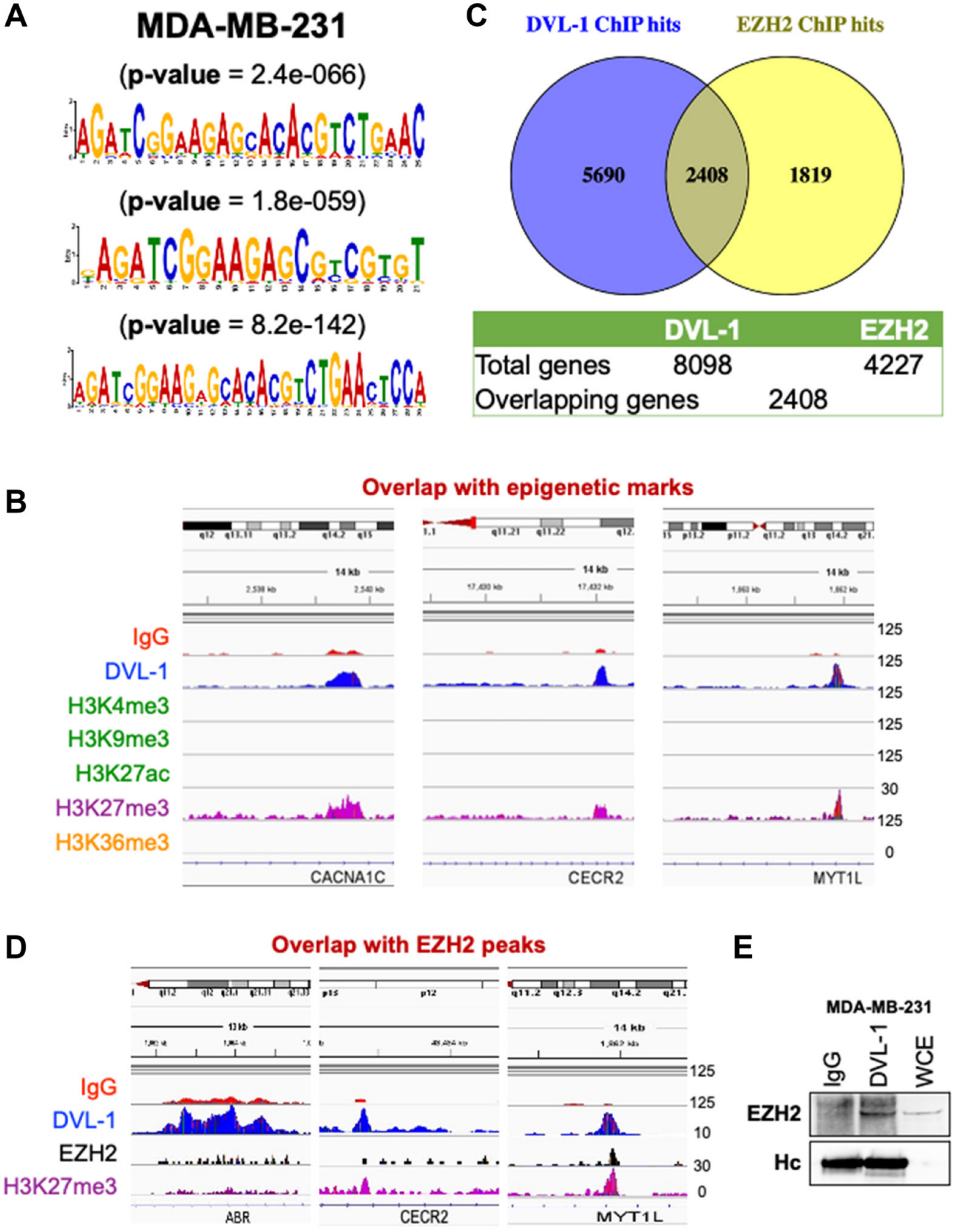
ChIP-Seq identified novel DVL-1 binding sequences and overlap with H3K27me3 peaks. (**A**) MEME-ChIP (Motif Analysis of Large Nucleotide Datasets) analysis of the DVL-1 binding sites as consensus motifs in MDA-MB-231 cells. (**B**) An assembly of IgG, DVL-1, H3K4me3, H3K9me3, H3K27ac, H3K27me3, and H3K36me3 ChIP-Seq in MDA-MB-231 for *CACNA1C, CECR2, and MYT1L* genes visualized by IGV. (**C**) Overlapping genes between DVL-1 and EZH2 (**D**) An assembly of IgG, DVL-1, EZH2, and H3K27me3 ChIP-Seq in MDA-MB-231 for *ABR, CECR2, and MYT1L* genes visualized by IGV. The last row show the location and orientation of DVL-1 direct target genes. (**E**) DVL-1 co-immunoprecipitates EZH2 in MDA-MB-231 cells. IgG heavy chain (Hc) was blotted for as a control for equal antibody loading for immunoprecipitation and whole cell extracts (WCE) as a positive control.

To identify the DNA motifs driving the association of DVL-1 to the genome, we analyzed the genomic DNA sequences from DVL-1 binding peaks detected by ChIP-Seq analyses. All the peaks were submitted to MEME-ChIP analysis to determine significant motifs that had a strong central enrichment among the DNA sequences for the ChIPs performed. MDA-MB-231 motifs were: AGATCGGAAGAGCACACGTCTGAAC (*p*-value = 2.4e-066), AGATCGGAAGAGCGTCGTGT (*p*-value = 1.8e-059), AGATCGGAAGAGCACACGTAACTCCA (*p*-value = 8.2e-142) (Figure 6A).

The negative correlation between DVL-1 and expression of its direct target genes, suggested that DVL-1 may facilitate gene silencing at distinct genes, therefore we assessed whether DVL-1 can complex with repressive epigenetic marks. Thus, we evaluated DVL’s genomic localization with H3K27me3, an epigenetic modification associated with transcriptional repression. Visualizing the genomic regions of some of the top DVL-1 hits such as *CACNA1C, CECR2,* and *MYT1L* in MDA-MB-231 cells (Figure 6B), we observed that DVL-1 peaks overlapped with the epigenetic mark H3K27me3. In contrast, there was an absence of active epigenetic marks – H3K4me3, H3K9me3, H3K27ac, and H3K36me3 at DVL-1 binding regions, perhaps indicating that DVL-1 might be localizing at genes that are silenced in MDA-MB-231 cells.

Intrigued by the role of DVL-1 in the trimethylation of H3K27, a hallmark of gene repression, we asked whether DVL-1 may act as a scaffold of any H3K27 methyltransferase implicated in H3K27 methylation. In our screen we identified EZH2 as a good candidate and investigated further. Interestingly, our data shows that DVL-1 and EZH2 co-localize at several regulatory regions. Using Venny tools, we observed an overlap of 2408 genes between DVL-1 peaks with published *EZH2* ChIP-Seq data **(**Figure 6C). Next, to determine if there is a link between DVL1-EZH2-H3K27me3, we first observed an overlap of the DVL-1 peaks with EZH2 at the genomic regions of our representative panel of novel DVL-1 targets such as *ABR, CECR2,* and *MYT1L* (Figure 6D). To further assess the significance of this finding, we also evaluated the physical interaction between DVL-1 and EZH2 by co-immunoprecipitation (Co-IP). Using Co-IP screen, we confirmed that EZH2 binds to DVL-1 in the MDA-MB-231 cell line (Figure 6E), indicating that DVL-1 and EZH2 may be part of a complex to regulate gene transcription. Altogether these data suggest that DVL-1 colocalizes with H3K27me3 and EZH2 at silenced regulatory regions of the chromatin.

### Conserved lysines K69 and K285 on DVL-1 influences regulation of multiple target genes critical for tumorigenesis

Since K69 and K285 residues are known regulatory switches for DVL-1 nuclear localization [6], we wanted to determine whether these K-residues on DVL-1 also influence transcript levels of DVL-1 target genes such as *TRIO, COL5A1* and *EXD3* implicated in cancer.

**Figure 7:**
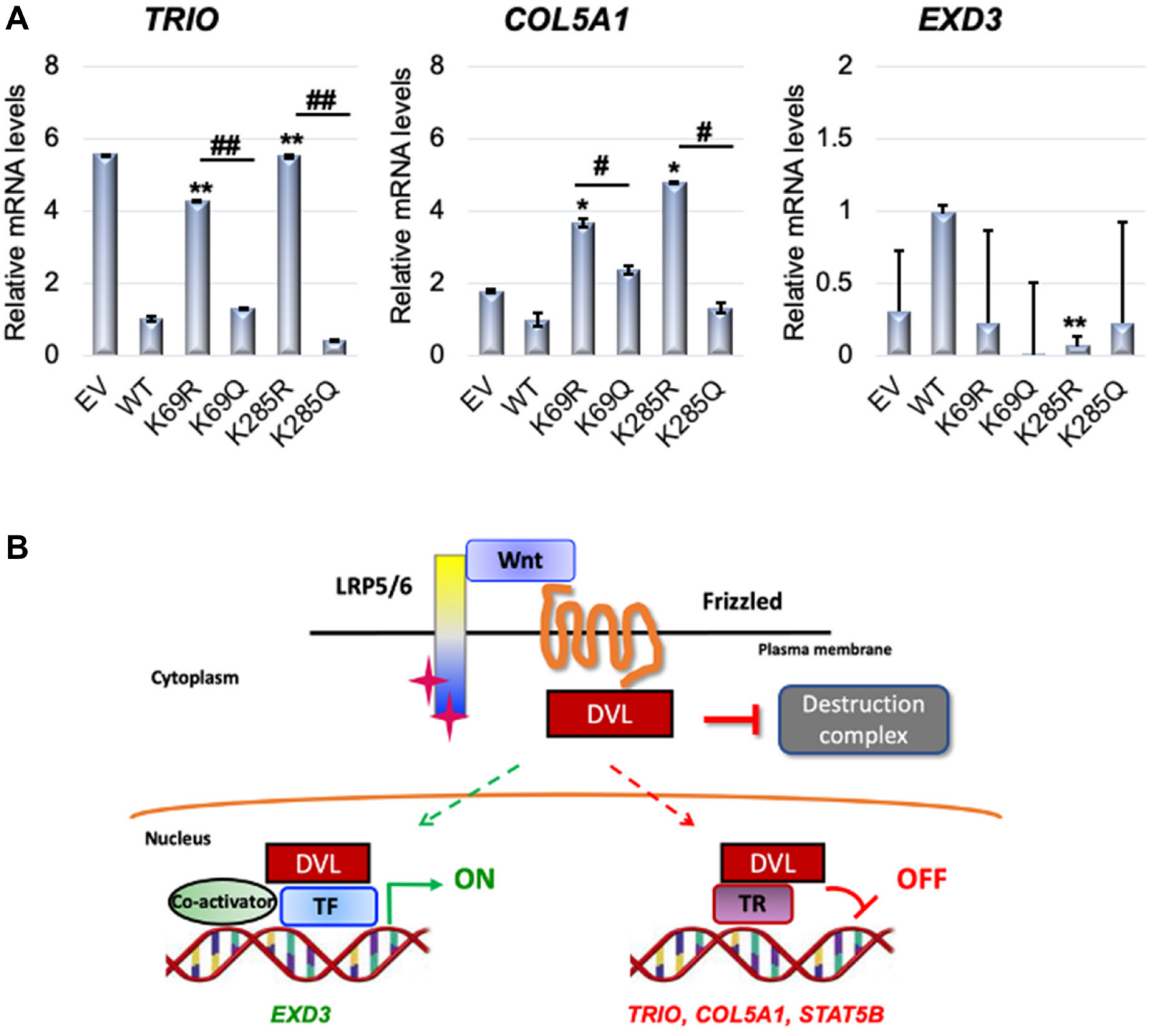
Conserved lysines on DVL-1 regulates target gene expression. (**A**) RNA was isolated from MDA-MB-231 cells stably expressing EV (empty vector), HA-tagged wild-type DVL-1 (WT), HA-tagged deacetylation mutants (K69R and K285R), and HA-tagged acetylation mutants (K69Q and K285Q) and cDNA was synthesized. Quantitative PCR was then performed using primers specific for *TRIO, COL5A1*, and *EXD3* transcript with primers in the coding region common to all transcripts. The sign “^*^” represents significant change in gene expression between WT and mutants, while “^#^” represents significant change between R and Q mutants. All results are expressed as mean ± SEM and were considered significant at ^*/#^*p* < 0.05, ^**/##^*p* < 0.01 and ^***/###^*p* < 0.001. (**B**) Schematic representation of the functional significance of DVL-1 in the nucleus as a transcriptional regulator by acting as a nuclear scaffold for transcription factors (TF) or repressors (TR) on various genes. Based on RNA expression analysis, DVL-1 seems to act as a transcriptional activator of *EXD3* genes, while DVL-1 seems to act as a transcriptional repressor on other genes such as *TRIO, COL5A1, and STAT5B.*

RNA was isolated from MDA-MB-231 cells stably expressing EV, WT-DVL-1, deacetylation mutants (K69R, K285R), as well as acetylation mutants (K69Q, K285Q). We used real-time qPCR with β-actin as an internal control to determine if WT versus mutants altered mRNA levels of several DVL-1 target genes that are critical for tumorigenesis. Interestingly, we found that WT, K69 and K285 acetylation mimetic (K69Q and K285Q) corresponded with a statistically significant decrease in the *TRIO* and *COL5A1* transcripts with respect to EV and deacetylation mutants (K69R and K285R) in MDA-MB-231 cells. Furthermore, we observed that there was a significant increase in transcript levels of *EXD3* in MDA-MB-231 cells with respect to K285R deacetylation mimetics (Figure 7A). These results show that nuclear DVL-1 and K-residues promoting its nuclear localization correlate with decreased gene expression, suggesting that nuclear DVL-1 is frequently linked with transcriptional repression of its target genes.

Altogether, we found that DVL-1 localizes at various transcriptional regulatory regions in breast cancer. As summarized in the model, DVL-1 binds to genomic regions and regulates transcription by potentially acting as a scaffold for transcription factors, suggesting a novel role of DVL-1 as a dynamic transcriptional regulator, both as an activator or repressor, based on the genomic location in the nucleus (Figure 7B).

## DISCUSSION

DVL proteins are an integral hub of the Wnt signaling pathway, that mediates oncogenic Wnt signals to downstream to propagate tumor cell proliferation, differentiation, and migration. Wnt signaling is notoriously hyperactivated in different tumor types such as gastrointestinal cancer, leukemia and breast cancer [3, 15, 18]. Alteration in DVL proteins have been reported for several cancer types. For instance, a study using glioma as a model, suggested that DVL expression may contribute to the malignancy and invasion of human glioma [19]. Very recently, nuclear DVL proteins have been proposed to play an essential role in promoting canonical Wnt pathway. Accumulation of DVL-2 in the nucleus has been shown to result in hyper-activation of Wnt signaling in colorectal cancer [20]. Another study reported that nuclear DVL has been linked to lymph node metastases and poor prognosis of non-small-cell lung carcinoma [21]. DVL-1 nuclear staining was observed in almost 54% of cases with a significant increase in nuclear β-catenin staining in lung carcinomas [22]. Likewise, other studies suggested the carcinogenic potential of DVL proteins in chronic lymphocytic leukemia and breast cancer [23, 24]. Amplification of DVL-1 was observed in 13 of 24 primary breast cancer patients in comparision to corresponding non-cancerous breast tissues, with a significant association in nuclear localization of DVL and β-catenin proteins [25].

While several studies suggest that DVL proteins could serve as a potential prognostic biomarker for breast cancer and other cancer types, the true potential of DVL-1 and its contribution to clinical factors which regulate breast tumor progression and tumor immunology remained unanswered. This study utilizes publicly available data, bioinformatics tools, and genomic analyses to emphasize the contribution of DVL-1 in the most aggressive subtype of breast cancer known as triple-negative breast cancer. We start with examining the expression levels of DVL-1 in breast cancer using bioinformatics analysis which indicates high DVL-1 expression in breast cancer compared to non-cancer breast tissues, specifically in luminal B and triple-negative tumors. Moreover, we found a correlation between DVL-1 and several risk-factors for breast cancer using UALCAN and bc-Gen ExMiner databases. While assessing few clinical parameters, we found that DVL-1 expression was significantly associated with cancer stages, menopausal status, and triple-negative status in breast cancer patients. Herein, we extensively investigate association between DVL-1 expression with breast cancer subtypes, clinicopathological parameters, and various risk factors associated with breast cancer. Interestingly, we also found that high expression of DVL-1 was associated with poor overall survival in basal or triple-negative breast cancer (TNBC), suggesting that the expression of DVL-1 could affect the prognosis of triple-negative breast cancer. In that line, we then extended our efforts to predict the correlation between DVL-1 expression and its role in modulating gene expression and immune cell infiltration in TNBC tumors. Here, we would like to note that while TNBC is often used as a surrogate for basal breast cancer and there can be significant overlap in gene expression profiles, the two tumor types can be biologically distinct. TNBC tumors are defined by a negative immunohistochemical staining for ER, PR, and HER2 receptor, while the basal subtype is defined by a distinct gene expression marked by strong expression of markers such as cytokeratins 5, 6 and 17 [26].

Few reports have investigated DVL nuclear localization, and little is known about the scope of its binding to genomic locations. In previous studies, we not only demonstrated that DVL translocates to the nucleus across multiple breast cancer cell lines but also that DVL-1 binds to multiple aromatase tissue-specific promoters and regulates their transcription [13]. We also showed that increase in DVL-1 localization at breast/adipose promoter (I.4) promoter resulted in significant decrease in the levels of I.4 and total aromatase transcript [6]. This important finding led us to pursue of a new gene targets which were not previously associated with the Wnt pathway. Using ChIP sequencing analysis, we have identified that DVL-1 localizes at over 8000 genomic loci in breast cancer models. These genes hits were significantly associated with pathways/functions such as developmental biology, GPCR signal transduction, endocrinology, ion channels, and metabolism of proteins. Interestingly, we found DVL-1 binding at various genes which have an important role in tumorigenesis and breast cancer metastasis.

The immune system can be activated by tumor antigens, and when primed can elicit antitumor response that results in tumor destruction. T-cell priming against tumor antigens followed by infiltration of the anti-tumor T cells is an important step to kill the tumor cells which leads to tumor control. However, often this response is hampered by several factors that can directly influence the efficacy of the immune response. Herein, our findings suggest that DVL-1 may be one of the factors that help the tumor cells to escape anti-tumor immune responses thereby allowing for the proliferation and growth of malignant cells. Wnt pathway is an important regulator of development and homeostasis of mammalian cells. Emerging reports suggest that Wnt signaling regulates T cell development and activation, suggesting that Wnt signaling controls immune regulation in mammalian system [27–30]. In addition, recent reports suggest that Disheveled 1 knockout (Dvl1^–/–^) mice causes dysfunction of Paneth cells along with an increase in CD8+ T cells in the gastrointestinal (GI) tract [31]. Moreover, recent clinical trials suggest that immune checkpoints inhibitors have reduced efficacy in TNBC patients that undergo neoadjuvant chemotherapy. Hutchinson et al. illustrates that the anti-tumor immune activity within tumor decreases during transition from primary to metastatic disease. Furthermore, they showed that TNBC progression was accompanied with decreased proportion of stromal tumor-infiltrating lymphocytes (TILs) including B cell, CD4+ naive T cell, CD8+ T cells and cancer-associated fibroblasts in metastatic TNBCs [32]. Here, we shed light on an important, yet unexplored aspect of DVL-1 expression and its association with the presence or absence of immune cell levels in breast cancer. Using genomic analysis (ChIP-Seq) we demonstrate that DVL-1 localizes to genes of several important immune cell genes in TNBC model. Furthermore, mRNA expression studies, demonstrate a negative correlation between DVL-1 expression and the markers for T cell exhaustion (e.g. CTLA4, HAVCR2), CD8+ T cell (CD8B), tumor-associated macrophages (TAM), T helper cell, and dendritic cells. Recent reports suggest that Wnt signaling promotes tumorigenesis by modulating tumor microenvironment by fine tuning the transformed cell and infiltrating immune cells [2]. Our analysis suggests that increased DVL-1 expression correlates with decreased expression levels of several immune cell genes and anti-tumor immune cell signaling regulators such as STAT5B. In addition, using RNA-Seq data from BC patients we observed a significant negative correlation between DVL-1 expression and CD8+ T cells markers, cDC1-recruting chemokines, T cell and NK cell-recruiting chemokines. Thus, these results could provide some clues as to why immune checkpoint inhibitors may be ineffective for breast cancer treatment where the levels of onco-proteins such as DVL-1 are relatively high.

Based on the classical Wnt model, cytoplasmic DVL-1 channels Wnt signals which activates canonical and non-canonical branches. However, in our genomic studies we noticed that nuclear DVL-1 seems to act as a transcriptional repressor localizing with histone methyltransferase EZH2 and a repressive H3K27me3 epigenetic mark at its target genes. We were also curious to understand whether Wnt target genes overlap with DVL ChIP hits. Based on the comprehensive ChIP Seq studies that we performed for DVL-1 and DVL-3 in breast cancer cell lines [33], we observed that DVL proteins target non-canonical genes. To further elaborate, we observed that only 9 DVL-1 ChIP hits overlap with Wnt target genes, namely *CD44, EGFR, FGF18, FGF4, LEF1, LGR5, MMP26, NOS2*, and *SALL4* genes. This might suggest a possibility that DVL proteins have functional implications beyond the classical Wnt model and also highlights the complexity of the signaling cascades in cancer context. Additionally, in the current study we report for the first time that DVL1 is associated with H3K27me3, a key repressive epigenetic mark that localizes to transcription regulatory regions, and we find an important overlap between DVL1 genomic occupancy and H3K27me3 peaks. Early studies by Kleer at al demonstrated that EZH2, a Polycomb Group (PcG) protein and transcriptional repressor involved in regulating cellular memory, was strongly associated with breast cancer aggressiveness. Overexpression of EZH2 in immortalized human mammary epithelial cell lines was shown to promote anchorage-independent growth and cell invasion [34]. Subsequent studies demonstrated that EZH2 overexpression led to aberrant terminal end bud architecture in the mammary gland and intraductal epithelial hyperplasia. In addition, EZH2 was found to physically interact with β-catenin and co-localize with β-catenin in human intraductal epithelial hyperplasia [35]. These early studies provided a link between EZH2 and β-catenin. The current study is the first to establish a link between EZH2 and DVL, a key regulator of both canonical and non-canonical Wnt signaling. While we have demonstrated a physical link and an overlap in occupancy of DVL1 and EZH2 at genomic loci, more questions remain to be addressed regarding the functional significance of their association. Additionally, others have reported that the Polycomb Repressive Complex 2 (PRC2), of which EZH2 is a part, establishes repressive H3K27me3 epigenetic signatures on genes involved in Wnt-governed intestinal homeostasis and Wnt-linked intestinal crypt dysfunction [36]. However, much more investigation is needed to decipher the functional significance of this convergence. Given these important links, we reason that DVL1 could be a key mediator that relays information initiated via Wnt ligands leading to epigenetic changes important for cellular and morphological phenotypes.

Collectively, our study demonstrates that high levels of DVL-1 is observed in breast cancer versus healthy tissue and is markedly associated with poor prognosis in triple-negative breast cancer. DVL-1 expression appears to negatively correlate with the presence of immune cells within the tumor microenvironment suggesting that DVL-1 could serve as suitable immune-modulating, prognostic biomarker for triple-negative breast cancer, a breast cancer type with unmet therapeutic needs.

## MATERIALS AND METHODS

### Tumor immune estimation resource data analysis

TIMER (Tumor IMmune Estimation Resource) is a web server for comprehensive analysis of tumor-infiltrating immune cells. Here we analyze the expression of DVL-1 in different cancer types, where tumor vs. normal sample types is plotted on x-axis and the DVL-1 gene expression is represented by log_2_ TPM. This resource is designed to systematically estimate tumor infiltrating cells such as B cells, CD4+ T cells, CD8+ T cells, neutrophils, macrophages and dendritic cells across diverse cancer types using a TIMER algorithm [37]. Moreover, this database can be used to explore the correlation between gene expression and abundance of immune infiltrates from RNA-seq expression profile. Correlation between DVL-1 and immune infiltration gene markers were explored using TIMER.

### Human protein atlas analysis

HPA (https://www.proteinatlas.org) database aims to map all human proteins in different cell types, tissues and organs using various omics techniques like antibody-imaging and mass spectrometry-based proteomics [38]. This resource compared protein levels between normal and cancer tissues using immunostaining. In our study, we determine the expression of DVL-1 proteins between normal and breast cancer tissues under pathology section in HPA database (Antibody: CAB011538).

### UALCAN analysis

UALCAN (http://ualcan.path.uab.edu) is a user-friendly web resource for analyzing OMICS data (TCGA and MET500) [39]. In this study, UALCAN was used to plot graphs depicting DVL-1 gene expression based on different clinical parameters in breast cancer carcinoma data set. The relative expression of DVL-1 was plotted based on parameters such as sample types (normal and primary tumor), breast cancer subclasses (normal, luminal, HER2 positive and TNBC), individual cancer stages (Stage 1, 2, 3, and 4), and menopausal status (normal, pre-menopause, peri-menopause, and post-menopause).

### Kaplan-Meier plotter database analysis

The Kaplan Meier Database (https://kmplot.com/analysis) is used to evaluate effect of genes on survival status in different cancer types. We compare the expression of DVL-1 and survival status in different molecular subtypes of breast cancer including luminal A (*n* = 2504), luminal B (*n* = 1425), HER2+ (*n* = 335), and TNBC group (*n* = 879). Overall survival (OS) were evaluated using Kaplan Meier plotter database. Hazard ratio (HR) for 95% confidence interval and significance value (p) less than 0.05 were considered statistically significant.

### Breast cancer GenExMiner 4.4 analysis

BC-Gen Ex Miner v4.4 (http://bcgenex.centregauducheau.fr/) statistical tool contains breast cancer (BC) transcriptome data (DNA microarrays and RNA-seq data) which allows gene expression analysis in breast cancer [40, 41]. This database contains three modules: correlation, expression, and prognosis. The correlation module identifies whether the gene-of-interest is either positively or negatively correlated with co-expressed genes located on the same chromosomal region. The expression analysis compares expression of genes to critical clinical parameters such as HER2, ER, PR status and other parameters. Lastly, the prognostic module allows the assessment of the prognostic impact of a gene on all possible combinations of population. For our study, we logged to the BC-Gen Ex Miner homepage, and selected RNA seq data and entered the DVL-1 as query gene. We then analyzed DVL-1 expression level based on various pre-determined clinical parameters such as nodal status (N+ positive or N− negative), estrogen receptor (ER+/ER−), progesterone receptor (PR+/PR−), HER2 status (HER2+/ HER2−), triple-negative status (TNBC and not-TNBC). The data is summarized as Table 1, where *p*-value less than 0.05 were considered statistically significant.

### Cell culture

All cell lines (MDA-MB-231, and MCF7) used in this manuscript were purchased from ATCC which utilizes STR technology for Cell Authentication, and they were used in a low passage (<20) within 6 months or less after receipt or resuscitation. MDA-MB-231 cells were cultured in DMEM (Gibco, 11995-065), while MCF7 cells were cultured in MEM (Gibco, 11095-080) supplemented with 0.1% insulin (Sigma, I0516). All culture media were supplemented with 10% fetal bovine serum and 1% penicillin/streptomycin (Gibco, 15070-063).

### Chromatin immunoprecipitation (ChIP), ChIP-Seq and data analysis

ChIP was done according to a published protocol [6, 13]. Briefly, cells were grown to confluence in 100 mm dishes; a final count of approximately 5 × 10^6^ cells per plate. Cells were cross-linked with 1% (w/v) formaldehyde for 8 min at room temperature and quenched in 0.125 M of glycine for 5 minutes at room temperature. The medium was removed, and cells were washed twice with PBS. Cells were scraped, pelleted and washed twice with PBS plus protease inhibitor cocktail. Cells were resuspended in SDS Lysis buffer (50 mM Tris-HCl pH 8.0, 10 mM 0.5 M EDTA, and 1% SDS) with protease inhibitor cocktail. Cells were then sonicated in a Diagenode Bioruptor sonicator for 30 cycles (30 sec pulses and 30 sec rest) and washed twice with chilled 1X PBS containing a protease inhibitor cocktail, scraped off the plates and collected by centrifugation. The soluble chromatin fraction was quantitated and 100 μg of chromatin was immunoprecipitated with anti-DVL1 antibody (D3570; Sigma), or Rabbit IgG (I5006; Sigma) for 2 h at 4°C. Dynabeads Protein A (Invitrogen, 10002D) were added to the chromatin-antibody mixture and incubated with rotation for 2.5 hours at 4°C. ChIPs were washed with five low salt wash buffer (0.1% SDS, 1% Triton X-100, 2 mM EDTA, 20 mM Tris HCl pH 8.1, and 150 mM NaCl), three high salt wash buffer (0.1% SDS, 1% Triton X-100, 2 mM EDTA, 20 mM Tris HCl pH 8.1, and 500 mM NaCl), and one TE wash (1 mM EDTA and 10 mM Tris HCl pH 8). Immunoprecipitated chromatin samples were reverse-crosslinked overnight at 65°C, followed by RNAse A (Promega) at 37°C for 2 hours, and proteinase K incubation (Promega) at 55°C for 2 hours. DNA was eluted using Qiaquick PCR purification kit (Qiagen) and amplified by end-point PCR and real time qPCR using gene-specific primers (Table 3A). DNA from ChIP was extracted, and at least 10 ng ChIP DNA was sent to GENEWIZ and used for ChIP-Seq library preparation and sequencing. FASTQ files were analyzed using DNASTAR’s Laser Gene software. Sequenced reads were aligned against the human genome and the peaks were visualized using integrative genomic viewer (IGV). MEME-ChIP was used to analyze DVL-1 binding motifs and TOMTOM to identify if those motifs were similar to known consensus sequences using the MEME Suite Programs http://meme-suite.org/index.html [42]. Venn diagrams to identify the overlapping genes were generated using the Venny tool [43].

**Table 3:** List of RNA primers and ChIP-primers used in the study

**Table 3A: List of ChIP primers**		
**Gene**	**Forward primer**	**Reverse primer**
*TRIO*	CAGGAGGCAAGCTTCTGATAGTA	CCACTACCTTTGTCAGTCAGCA
*COL5A1*	TGAGTTTGGTTCTCGGGTTCC	AGGAGGACACCCACCCATAC
*EXD3*	AGCCTGTACCCTCCTGAGAT	GGTGAGTGCTGAGTCCAAGT
*OR4A47*	GGCAAAAAGCCCTCTCAACC	TGACTCTCTTCCTGGTGGGTA
*GLDN*	CCCACATTCCCGTTGGTGTT	CTGAGGTCAGACACCCTGGA
*EFCAB6*	GAACCAAGCAGGAAGGAACTG	ATGTCCAGCTTTTGGCAGCTA
*CD8B*	GGGCCTACCTCCAAAGATGC	GGTTGGTGTGGGGCAGATTA
*CCR8*	AAGAAGGGCATGAGTAGGCA	CATGCATGTTTGTGGGCTCT
*CD1C*	GCTTAGTGGGAGAGCAGCTA	GACAATCTTCCCCATCAGCCA
*MS4A4A*	GGTCACAAGAGGGGTGGAAC	AAGCTCGGCTACTTCAGCAC
*STAT5B*	TCCTGTGTGCCTCACCAATC	CCACCCTCCCTGGATAATGC
*STAT4*	AGCATTGGAGTGGTAGCAGG	GCTGGAGATGTGGTCCCTTT

**Table 3B: List of RNA primers**		
**Gene**	**Forward primer**	**Reverse primer**
*TRIO*	TTCTTCCGATCCGGGTTTCG	AGACCTCCTCGCTGGGAATA
*COL5A1*	AGCAGCCTTCCACCTGGTAT	GGTAGGTGACGTTCTGGTGG
*EXD3*	CTGCCAGGCCTGTAACTGTG	CCGTCCCAGAAGACCTTTCC
*STAT5B*	GCCGAGAAGCACCAGAAGACC	CGGCCAGCATCTCCTCCA
*BACTIN*	GGACTTCGAGCAAGAGATGG	AGCACTGTGTTGGCGTACAG

### The Cancer Genome Atlas (TCGA) data analysis

Genomic data from Breast Cancer (METABRIC, Nature 2012 & Nat Commun 2016) study was analyzed using cBio Cancer Genomics Portal (http://cbioportal.org). The study consisted of gene expression analysis from 1904 breast cancer patient [44]. Correlation analysis was conducted to determine genes which were common between genes altered in breast cancer patients and DVL-1 ChIP-Seq analyses in MDA-MB-231 and MCF7 cells.

### Stable expression of WT and mutants in MDA-MB-231 cell lines

350,000 cells were plated in a p6-well dish. After 24 hours, cells were transfected with 1 μg DNA of empty vector (pcDNA3.1(+) without HA-tag), full-length DVL-1 (WT), K to R, K to Q point-mutants using lipofectamine 3000 reagent (Thermo Scientific). After 24 hours, the cells were selected using 1 mg/ml G418 antibiotic until no cells were remaining in non-transfected control.

### DVL-1 stable knock-down

MDA-MB-231 were infected by pLKO.1-puro based shRNA MISSION lentiviral transduction particles for DVL-1 (TRCN0000441114, Sigma), and Non-Target shRNA control transduction particles (SHC002V, Sigma). 24 h prior to transduction, cells were plated in order to reach 80% confluency at the time of transduction. The transduction was enhanced with Hexadimethrine Bromide (Sigma, H9268) at a final concentration of 8 μg/ml. Following the addition of hexadimethrine bromide, the appropriate amount of viral particles were added at 2× multiplicity of infection (MOI) to the media, which was replaced with fresh media after 24 h. The puromycin selection was started 48 h after transduction at a concentration 1 μg/ml of puromycin (Gibco, A11138-03), and the puromycin-containing media was replaced every 3–4 days until total selection was achieved.

### mRNA expression analysis

Total RNA was isolated from cells using a Bio-Rad RNA extraction kit. 2 μg of total RNA was reverse-transcribed using SuperScriptIII Reverse Transcriptase (ThermoFisher) to synthesize first-strand of complementary DNA (cDNA). Intron-spanning primers were designed for each specific target DNA and gene expression measured by either endpoint-PCR or real-time qRT-PCR (Table 3B).

### End-point PCR and quantitative real-time qRT-PCR

End-point PCR amplification was performed using JumpStart RedTaq (Sigma). The Applied Biosystems Veriti 96-well thermal cycler (Applied Biosystems) and Gel DOC EZ imager (Bio-Rad) were used analyses. Gene expression was quantified by real-time qPCR in CFX96 Normal-Well Real-Time System (BioRad) using PerfeCta SYBR Green FastMix and specific oligonucleotide primers. The reaction mixtures contained 10 μl PerfeCta SYBR Green FastMix, 7.2 μl ddH_2_O, 2.0 μl template cDNA and 0.4 μl gene-specific 10 μM PCR oligonucleotides primers. The reaction conditions were 95°C for 30 s, followed by 40 cycles of 95°C for 5 s and 60°C for 30 s and Melt Curve (dissociation stage). Relative gene expression was calculated as delta (Δ Re (the difference between the cycle threshold values, Ct, of the internal control, and Ct of gene of interest) and confirmed by 2–ΔΔ CT method.

### Co-immunoprecipitation

Cells were seeded and incubated at 37°C under conventional O_2_ conditions. Once they were 70% confluent, cells were washed with PBS and lysed in Co-IP lysis buffer (25 mM Tris, pH 7.4, 150 mM NaCl, 1 mM EDTA, 1% NP-40, 5% glycerol and Protease inhibitor cocktail). This was followed by quantification for equal protein loading using standard BCA protocol (Thermo Scientific, 23227). Cell extracts were incubated with 2 μg of DVL-1 specific antibody (D3570; Sigma), and species-matched IgG as a negative control for 2 hours/4°C. Protein A Dynabeads (Life Technologies) were incubated with the antigen-antibody complex for 2 hours at 4°C. Beads were washed four times with lysis buffer with gentle agitation for 5 minutes per wash. 5× sample buffer (Invitrogen) was used for elution of complex from beads followed by Western blotting along with WCE.

### Immunoblots

Samples were subjected to polyacrylamide gel electrophoresis using Invitrogen Nupage gel system, transferred to PVDF (Millipore) membranes, and immunoblotted. Antibodies used are as follows: EZH2 (cst-5246; Cell Signaling). Membranes were incubated in 5% BSA/TBST with primary antibody overnight at 4°C. Membranes were washed with TBST and probed with horseradish peroxidase-conjugated secondary antibodies in 5% BSA/TBST for 1 hour/room temperature (RT). Membranes were visualization by enhanced chemiluminescence (ECL) reagent (Thermo Scientific) using Azure C300 gel imaging system (Azure Biosystems).

### Statistical analysis

The difference in expression of DVL-1 gene in different samples types, cancer subclasses, and clinical parameters were analyzed using UALCAN and BC-GenExMiner database. Moreover, the difference between high and low expression of DVL-1 in survival curves were obtained using Kaplan-Meier method. The results from these databases were with *p*-value and log-hazard ratios (later for survival curves only) from a log-rank test. The Pearson correlation coefficient was used to measure any correlation among genes, where 0 = |r| represents no correlation, 0.0 < |r| < 0.2 is weak correlation, 0.2 ≤ |r| < 0.4 is low moderate correlation, 0.4 ≤ |r| < 0.6 is moderate correlation, 0.6 ≤ |r| ≤ 0.8 is strong correlation, 0.8 ≤ |r| < 1.0 represents very strong correlation, and 1.0 = |r| : perfect correlation. The same is true for negative correlation. Lastly, *p*-value (p) less than 0.05 was considered statistically significant.

## SUPPLEMENTARY MATERIALS


