# Supplementary material for: Genomic profiling of DVL-1 and its nuclear role as a transcriptional regulator in triple negative breast cancer
Source: Genes Cancer. 2021 Oct 13;12:77–95. doi: 10.18632/genesandcancer.217 (PMC8513712; doi:10.18632/genesandcancer.217)
Supplement: Supplementary file 1 [file ganc-12-217-s001.pdf]

Genomic profiling of DVL-1 and its nuclear role as a transcriptional regulator in triple negative breast cancer

SUPPLEMENTARY MATERIALS

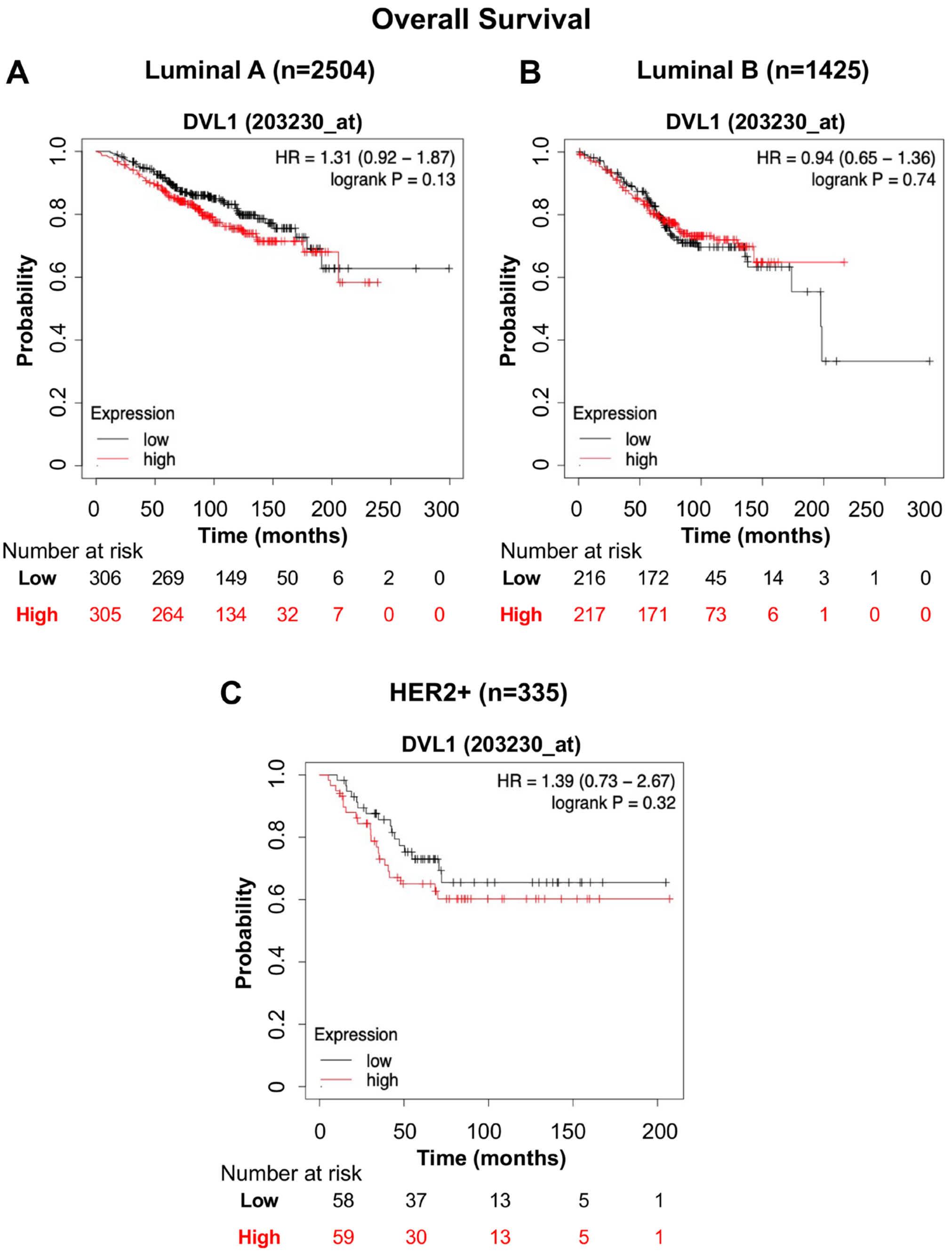

Supplementary Figure 1: Association of DVL-1 expression with overall survival (OS) in different breast cancer subtypes. Kaplan Meier database was used to compare survival rates between high and low expression of DVL1 (203230_at) in different subclasses of breast cancer. Survival curves of OS and DMFS in (**A**) Luminal A (*n* = 2504), (**B**) Luminal B (*n* = 1425), and (**C**) HER2+ (*n* = 335).

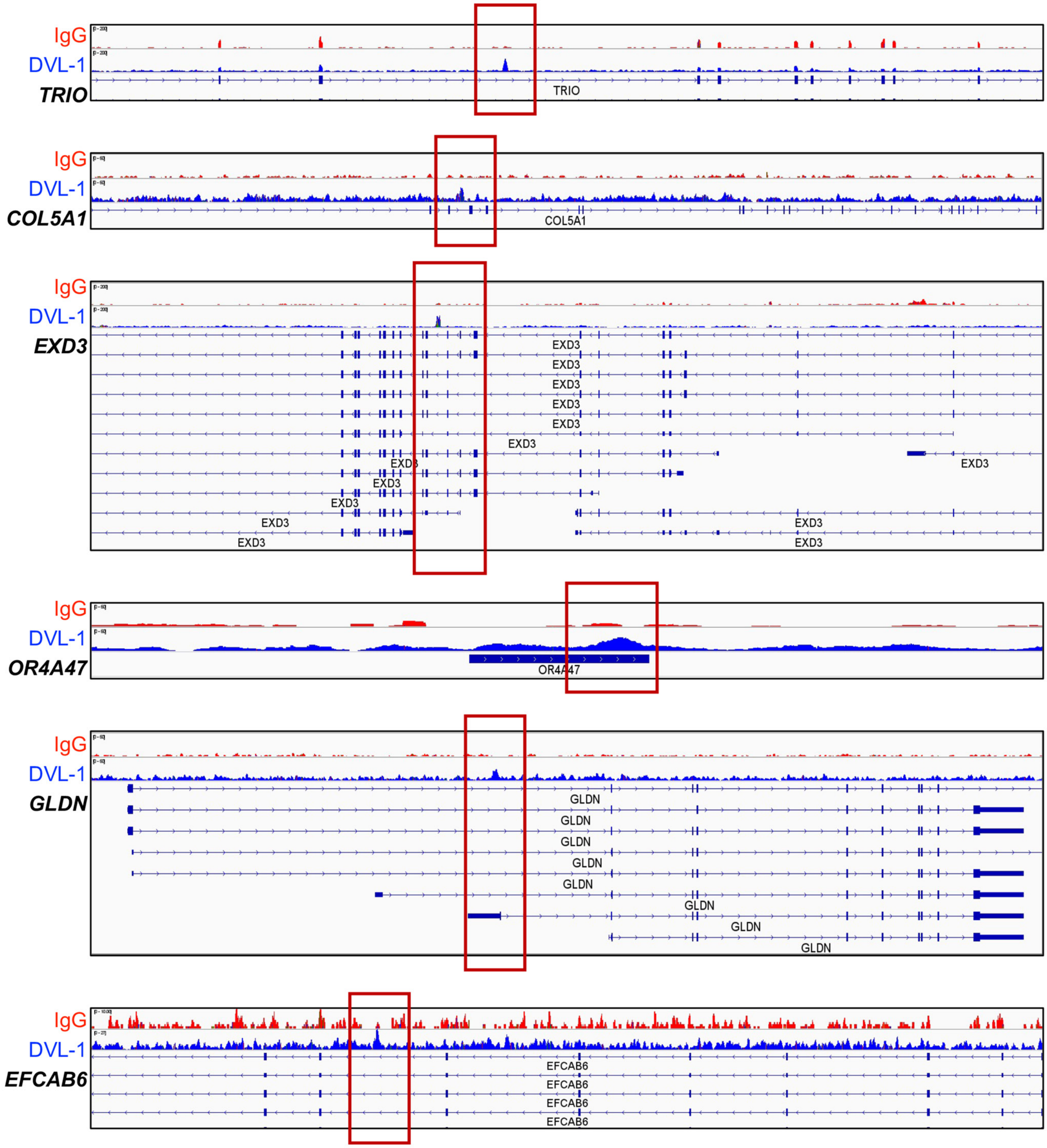

Supplementary Figure 2: DVL-1 localizes at various cancer-associated genes in MDA-MB-231 cells. An assembly of IgG (first row) and DVL-1 (second row) ChIP-Seq data in MDA-MB-231 for the *TRIO, COL5A1, EXD3, OR4A47, GLDN*, and *EFCAB6* genes, visualized by IGV. The true peaks representing enrichment of DVL-1 at a specific gene location is highlighted within the box.

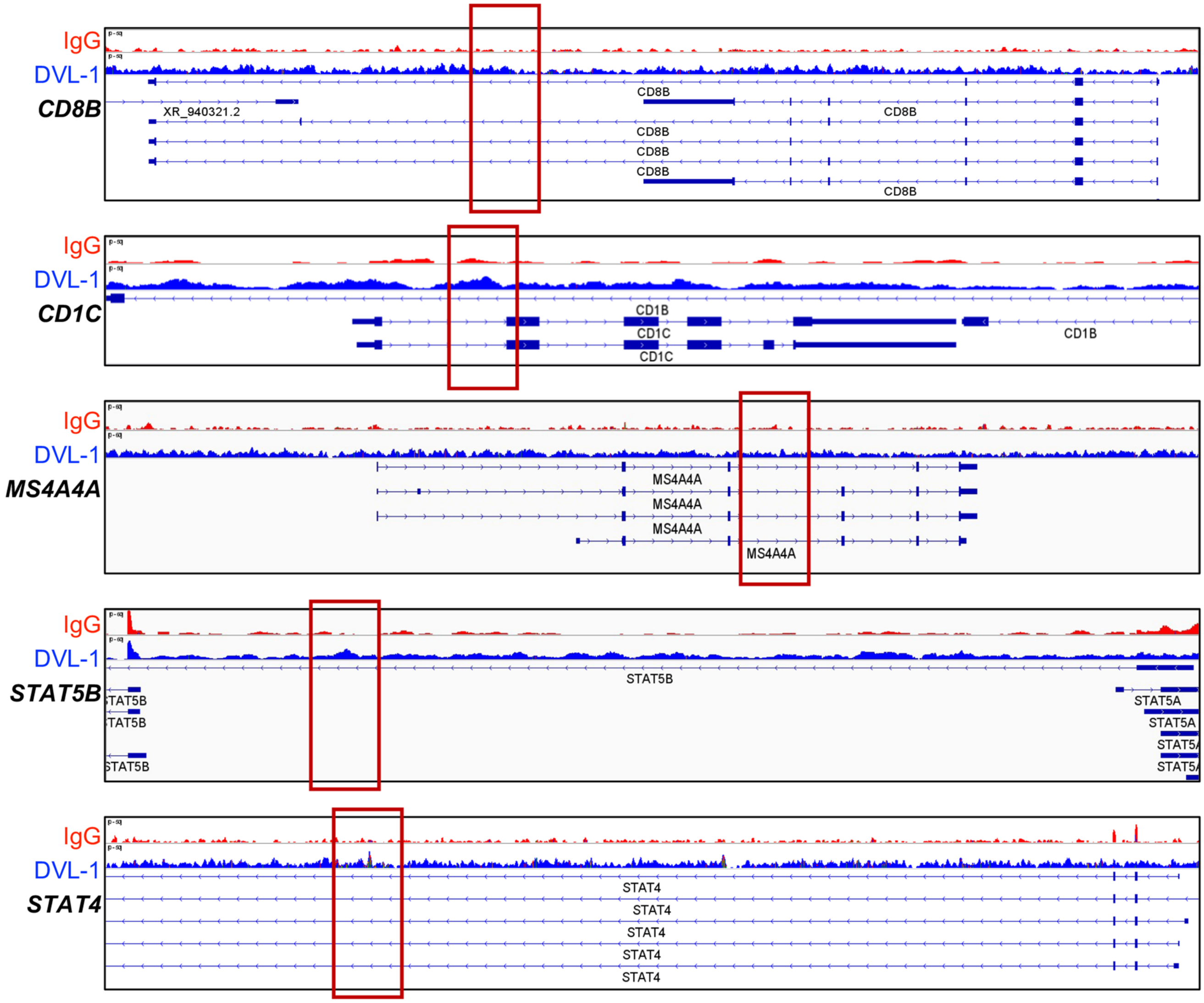

Supplementary Figure 3: DVL-1 localizes at immune cell genes in MDA-MB-231 cells. An assembly of IgG (first row) and DVL-1 (second row) ChIP-Seq data in MDA-MB-231 for the *CD8B, CD1C, MS4A4A, STAT5B, and STAT4* genes, visualized by IGV. The true peaks representing enrichment of DVL-1 at a specific gene location is highlighted within the box.

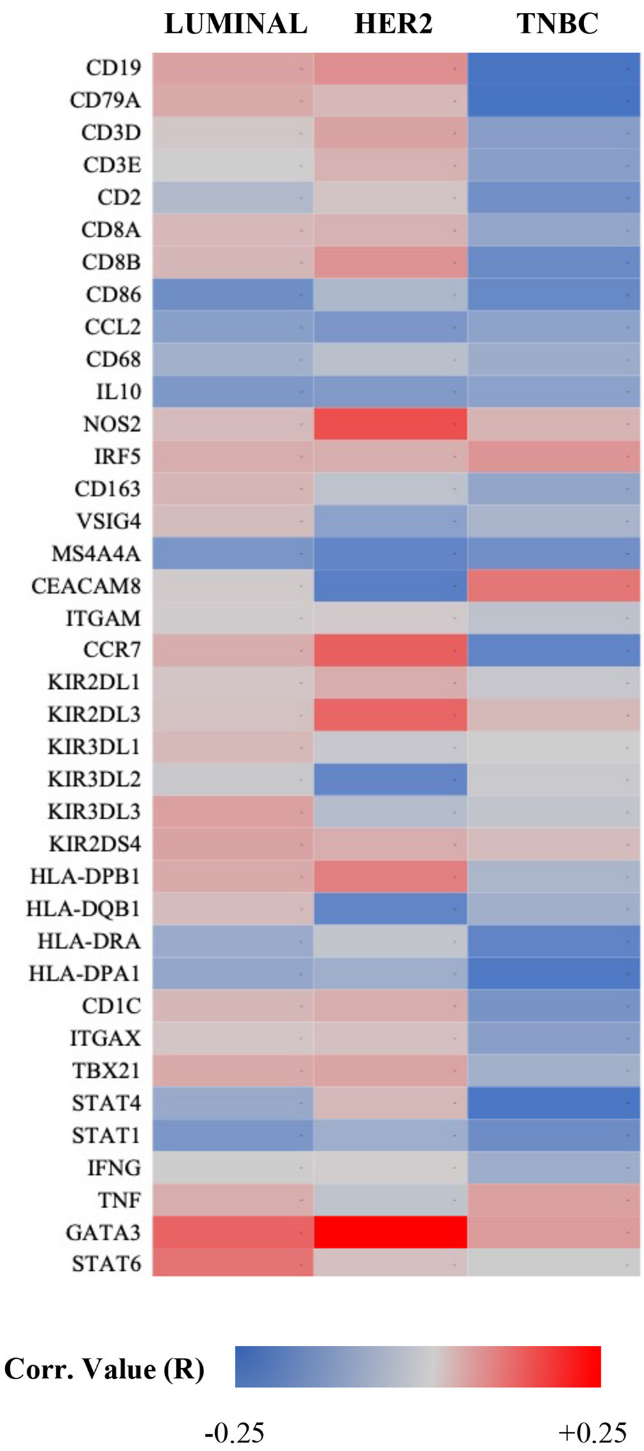

Supplementary Figure 4: Heatmap representing correlation between DVL-1 expression and markers of immune cells in breast cancer. (Source: Table 2).
